# Transcriptome Analyses of Adipose Tissue Samples Identify *EGFL6* as a Candidate Gene Involved in Obesity-Related Adipose Tissue Dysfunction in Children

**DOI:** 10.3390/ijms23084349

**Published:** 2022-04-14

**Authors:** Kathrin Landgraf, Andreas Kühnapfel, Maria Schlanstein, Ronald Biemann, Berend Isermann, Elena Kempf, Holger Kirsten, Markus Scholz, Antje Körner

**Affiliations:** 1 Center for Pediatric Research Leipzig (CPL), Hospital for Children & Adolescents, Leipzig University, 04103 Leipzig, Germany; maria.schlanstein@medizin.uni-leipzig.de (M.S.); elena_kempf@gmx.de (E.K.); antje.koerner@medizin.uni-leipzig.de (A.K.); 2 Institute for Medical Informatics, Statistics and Epidemiology (IMISE), Leipzig University, 04107 Leipzig, Germany; andreas.kuehnapfel@imise.uni-leipzig.de (A.K.); holger.kirsten@imise.uni-leipzig.de (H.K.); markus.scholz@imise.uni-leipzig.de (M.S.); 3 Institute of Laboratory Medicine, Clinical Chemistry and Molecular Diagnostics, University Hospital Leipzig, Leipzig University, 04103 Leipzig, Germany; ronald.biemann@medizin.uni-leipzig.de (R.B.); berend.isermann@medizin.uni-leipzig.de (B.I.); 4 LIFE Research Center for Civilization Diseases, Leipzig University, 04103 Leipzig, Germany

**Keywords:** adipose tissue, adipocyte, adipocyte hypertrophy, adipose tissue dysfunction, children, obesity, metabolic disease, transcriptome, *EGFL6*

## Abstract

Obesity develops early in childhood and is accompanied by early signs of adipose tissue (AT) dysfunction and metabolic disease in children. In order to analyse the molecular processes during obesity-related AT accumulation in children, we investigated genome-wide expression profiles in AT samples, isolated adipocytes, and stromal vascular fraction (SVF) cells and assessed their relation to obesity as well as biological and functional AT parameters. We detected alterations in gene expression associated with obesity and related parameters, i.e., BMI SDS, adipocyte size, macrophage infiltration, adiponectin, and/or leptin. While differential gene expression in AT and adipocytes shared an enrichment in metabolic pathways and pathways related to extracellular structural organisation, SVF cells showed an overrepresentation in inflammatory pathways. In adipocytes, we found the strongest positive association for epidermal growth factor-like protein 6 (*EGFL6*) with adipocyte hypertrophy. *EGFL6* was also upregulated during in vitro adipocyte differentiation. In children, *EGFL6* expression was positively correlated to parameters of AT dysfunction and metabolic disease such as macrophage infiltration into AT, hs-CRP, leptin levels, and HOMA-IR. In conclusion, we provide evidence for early alterations in AT gene expression related to AT dysfunction in children and identified *EGFL6* as potentially being involved in processes underlying the pathogenesis of metabolic disease.

## 1. Introduction

Over the last 40 years, obesity has reached epidemic proportions with more than 1.9 billion (i.e., 39%) overweight adults worldwide, and more than 650 million (i.e., 13%) being classified as obese in 2016 [1]. However, obesity is not a phenomenon amongst adults only, but it starts early in life. According to the World Health Organization (WHO), over 340 million children and adolescents aged 5 to 19 years were overweight or obese in 2016, and 39 million children under the age of 5 years were overweight or obese in 2020 [2]. Obesity is defined by excessive accumulation of adipose tissue (AT) due to the enlargement of adipocytes (hypertrophy) and/or formation of new adipocytes (hyperplasia) through proliferation and differentiation of adipose progenitor cells [3]. Especially, the hypertrophic AT phenotype is closely associated with the formation of AT dysfunction by triggering low-grade chronic inflammation, insufficient angiogenesis, and excessive collagen deposition in AT [4], which collectively lead to dysbalanced adipokine release, impaired glucose metabolism and, finally, to obesity-associated metabolic disease in adults including cardiovascular diseases [5], type 2 diabetes [6], non-alcoholic fatty liver disease [7], and cancer [8].

We have shown recently that the age of 2 to 6 years is a critical period for the development of sustained obesity and that almost 90% of children with obesity at the age of 3 years will remain overweight or obese in adolescence [9]. Moreover, the AT of children with obesity is characterised by early signs of AT dysfunction including adipocyte hypertrophy, AT inflammation, and alterations in cellular function such as lipolysis and adipokine secretion [3]. Importantly, the link between adipocyte hypertrophy and impaired glucose metabolism is already evident in children [3,10]. Hence, studies in children might provide valuable insights into the early processes involved in obesity-related AT expansion and, particularly, in the development of adipocyte hypertrophy and AT dysfunction. This could contribute to the identification of candidate targets for prevention and/or treatment of obesity-associated metabolic diseases.

The aim of the present study was to analyse the molecular processes during obesity-related AT accumulation and the development of adipocyte hypertrophy and AT dysfunction in children. Particularly, we investigated genome-wide expression profiles in AT samples, isolated adipocytes, and stromal vascular fraction (SVF) cells from lean children and children with obesity and assessed their relationship to obesity state as well as biological and functional AT parameters.

## 2. Materials and Methods

### 2.1. Human Adipose Tissue Biopsies (Leipzig Adipose Tissue Childhood Cohort)

The AT samples were obtained during elective surgery (i.e. orthopaedic surgery, herniotomy or orchidopexy, abdominal surgery, back surgery, and mammary reduction surgery) from 317 European children. Children with diabetes, generalised inflammation, cardiovascular or peripheral artery disease, malignant disease, or genetic syndromes were excluded from the study. Written informed consent was obtained from all parents. The study was approved by the local ethics committee (Reg. No: 265-08, 265-08-ff, University of Leipzig; NCT02208141). Body mass index (BMI) data were translated into a standard deviation score (SDS) based on age- and sex-specific German reference data [11]. Fasting serum adiponectin and leptin levels in blood were measured by a certified laboratory (Institute of Laboratory Medicine, Clinical Chemistry and Molecular Diagnostics, University of Leipzig). Adipocytes and SVF cells were freshly isolated from AT by collagenase digestion as previously described [3]. Adipocyte size was determined after fixation of freshly isolated adipocytes in osmium tetroxide (Science Services, Munich, Germany) using a Coulter counter (Multisizer III; Beckmann Coulter, Krefeld, Germany). The number of macrophages in AT was assessed by CD68 immunostaining (M0718, DAKO) using the DAKO REAL™ APAAP Immunocomplex system in paraffin-embedded tissue sections and is given per 100 adipocytes.

### 2.2. Metabolic Syndrome, Weight Loss Cohort

The subcutaneous AT of male individuals with metabolic syndrome was obtained by fine needle-aspiration from the periumbilical area before and after 6 month long telemonitored lifestyle-induced weight loss at the Institute of Clinical Chemistry and Pathobiochemistry, Otto-von-Guericke University, Magdeburg, Germany, in a prospective controlled clinical trial (ICTRP Trial Number: U1111-1158-3672). Participants were advised to lower their caloric intake by 500 kcal/day, to perform a low-carbohydrate diet with preference for low-GI carbohydrates, and to increase their usual daily physical activity but to keep their pulse below 120/min [12,13,14]. For gene expression analyses, 43 paired AT sample sets with high RNA quality were selected. Details about RNA isolation, microarray analysis, and statistical analyses have previously been described [13].

### 2.3. In Vitro Adipocyte Differentiation

Simpson–Golabi–Behmel syndrome (SGBS) cells were kindly supplied by Martin Wabitsch, University of Ulm, Germany [15], and were cultured in DMEM/Ham F12 medium (Thermo Fisher Scientific, Darmstadt, Germany) supplemented with 33 μmol/L biotin and 17 μmol/L pantothenic acid. Adipocyte differentiation was performed in four technical replicates and induced by treating confluent cells with serum-free medium containing 20 nM insulin, 100 nM hydrocortisone, 0.2 nM triiodothyronine, and 0.13 nM apo-transferrin. For the first 4 days of differentiation, 25 nM dexamethasone, 500 µM isobutyl-1-methylxanthine, and 2 µM rosiglitazone were added to the medium [16].

Cell culture and adipogenic induction of primary cells from adult patients (N = 5; Lonza, Cologne, Germany) and cryopreserved aliquots of SVF cells from children included in the Leipzig AT childhood cohort (N = 7) was performed according to the Poietics™ human adipose-derived stem cell–adipogenesis protocol (Lonza). For SGBS cells and primary cells from adults, analyses were performed at different time points of adipocyte differentiation from day 0 (before adipogenic induction) until day 12. For primary SVF cells from children, measurements before (day 0) and at day 8 of adipocyte differentiation were available.

### 2.4. RNA Isolation and cDNA Synthesis

Total RNA from AT, isolated adipocytes, and SVF cells as well as SGBS cells and primary cells from adults and children during in vitro adipocyte differentiation was isolated as previously described using the RNeasy Mini Kit (Qiagen, Hilden, Germany) including on-column DNA digestion according to the manufacturer’s instructions [17]. Pooled RNA samples from human tissues were obtained from Clontech (Takara Bio, Saint-Germain-en-Laye, France). An aliquot of 500 ng of RNA was reverse transcribed into cDNA using M-MLV Reverse Transcriptase (Thermo Fisher Scientific, Darmstadt, Germany).

### 2.5. Microarray-Based Transcriptome Analysis

Measurement of global gene expression in AT, isolated adipocytes, and SVF cells of children was performed with Illumina HumanHT-12 v4 BeadChip arrays. Imputation and background correction were performed with Illumina software, GenomeStudio Gene Expression Module v1.6+. Quality control of gene expression data was performed using the R package “HT12ProcessoR” developed by the working group of Markus Scholz, University of Leipzig, Germany, and is available on GitHub (https://github.com/holgerman/HT12ProcessoR, accessed on 31 July 2019) [18]. Descriptive statistics for probands and samples included in Illumina transcriptome and subsequent statistical analyses are given in Table 1.

### 2.6. Quantitative Real-Time RT-PCR Analysis

Quantitative real-time RT-PCR (qRT-PCR) was performed using the ABI 7500 Real-Time PCR System (Applied Biosystems, Darmstadt, Germany). Copy numbers of EGF-like domain multiple 6 (*EGFL6*) were determined from a standard curve and normalised to the mean of the reference genes beta-actin (*ACTB*), TATA-box-binding protein (*TBP*), and hypoxanthine phosphoribosyltransferase 1 (*HPRT*). For analysis, *EGFL6* expression was normalised to beta-actin (*ACTB*) and TATA-box-binding protein (*TBP*). Cohort characteristics of probands and samples included in the qRT-PCR analysis are given in Table 2. The primer and probe sequences used for the qRT-PCR are listed in Table S1.

### 2.7. Statistical Analyses

All analyses were performed in R using the packages “limma”, for linear modelling and to calculate moderated test statistics applying the empirical Bayes method, and the package “fdrtool” for controlling the false discovery rate (FDR).

Association analyses of Illumina transcriptome profiles with parameters of childhood obesity (BMI SDS; Table S2) and AT dysfunction (adipocyte size, macrophage infiltration, serum adiponectin, and leptin; Table S3) were performed separately using linear models adjusting for sex and age. These two basic adjustment variables, themselves, were of less interest in this study but were considered to determine the effect of the corresponding parameter as accurately as possible. For analysis of Illumina transcriptome data of primary SVF cells from children before and after adipocyte differentiation, we applied a linear mixed model to consider multiple measurements. The results can be found in Table S4. We used the beta estimates from linear (mixed) model analyses as effect sizes of interest. After performing analyses of cross-sectional and longitudinal data, gene expression results of adipocyte size and adipocyte differentiation were combined to a common candidate list for functional analysis, i.e., both sets of significantly regulated transcripts were intersected.

For pathway enrichment, we performed gene ontology (GO) pathway analyses of biological processes using the Web-Based Gene Set Analysis Toolkit (WebGestalt, http://www.webgestalt.org/, accessed on 11 November 2021) [19]. For this purpose, from all significantly regulated transcripts with an FDR less or equal to 5% for BMI SDS association or 20% for association with adipocyte size and adipocyte differentiation, the 250 respective transcripts with the highest absolute effect sizes were selected.

Statistical analyses of qRT-PCR data on human tissues, in vitro adipocyte differentiation experiments, and on isolated adipocytes and SVF cells of children were performed using GraphPad Prism 6 (GraphPad Software, San Diego, CA, USA) and Statistica 10 (StatSoft, Tulsa, OK, USA). Data that did not adhere to a Gaussian distribution were log-transformed to meet the required statistical assumptions before analyses. Parametric tests (i.e., Pearson correlation analysis, two-sided Student’s *t*-test, and one-way ANOVA) were applied for quantitative traits and chi-squared test for categorical traits, i.e., sex and occurrence of crown-like structures in adipose tissue. Patients with overweight and obesity were combined into one group.

## 3. Results

### 3.1. Obesity-Associated Alterations in Gene Expression Profiles in the Adipose Tissue of Children

**Figure 1:**
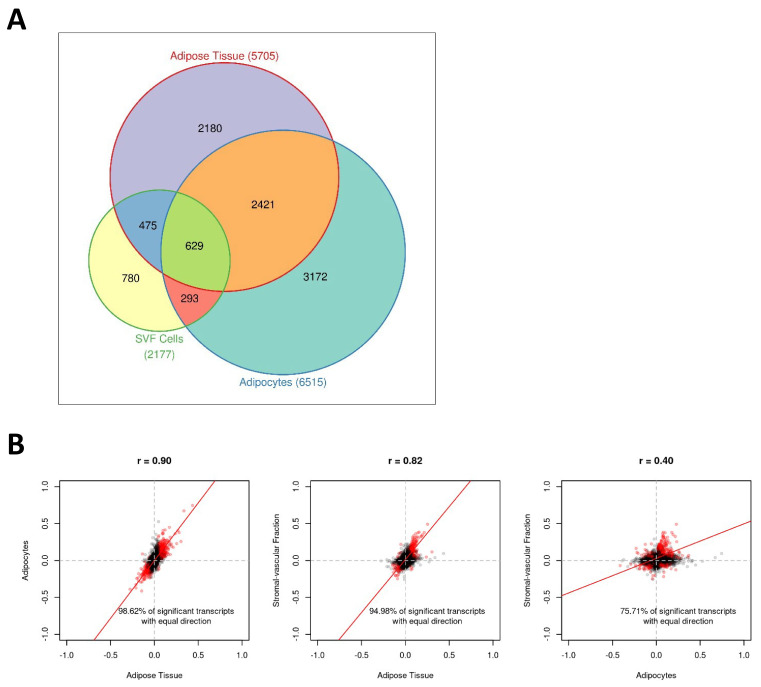
Childhood obesity is linked to alterations in gene expression profiles in adipose tissue. (**A**) Venn diagram displaying the number of significant (False discovery rate FDR ≤ 5%) transcripts for each tissue (i.e., adipose tissue, adipocytes, and stromal vascular fraction (SVF) cells). The numbers in brackets after the labels correspond to the absolute number of significant transcripts for the respective tissues. (**B**) Scatter plots of pairwise comparisons of the effect sizes between tissues. Transcripts expressed in all tissues (M = 20,664) were considered. Common significant transcripts (FDR ≤ 5% for each tissue) are red coloured. The regression line was estimated on the basis of the respective effect sizes.

In order to analyse the effect of BMI SDS on processes in the subcutaneous white adipose tissue (AT) of children, we performed gene expression analyses adjusted for age and sex in whole AT samples (N = 306). We identified 5705 significantly (FDR ≤ 0.05) regulated transcripts (i.e., 22.9% of all analysed transcripts) with 3054 transcripts being upregulated and 2651 transcripts being downregulated with increasing BMI SDS of children (Table S2). Analysing isolated adipocytes (N = 117) and cells of the SVF (N = 113) as AT subcomponents separately, we observed a comparable number of significantly regulated transcripts for adipocytes with 6515 transcripts (30.0% of all analysed transcripts), while SVF cells showed a lower proportion of significantly regulated transcripts (2177 transcripts, 9.3% of all analysed transcripts) (Table S2). In line with this, the strongest overlap of regulated transcripts was found between AT and adipocytes with 3050 transcripts detected as significantly regulated with BMI SDS in both tissues (Figure 1A). Comparison of the effect sizes showed a similarly high correlation between regulated transcripts of AT and adipocytes (*r* = 0.90) and AT and SVF (*r* = 0.82), which was not surprising given that adipocytes and SVF cells are subcomponents of AT. In contrast, the correlation of effect sizes between adipocytes and SVF was relatively low (*r* = 0.40), indicating that BMI-dependent gene expression changes affect different biological processes in these tissues (Figure 1B). This was further underlined by the results from the gene ontology (GO) pathway analyses showing that AT and adipocytes shared an enrichment of pathways related to extracellular structural organisation and receptor-mediated endocytosis, and they also had a particular overrepresentation of different metabolic pathways. In contrast, SVF cells presented a specific overrepresentation of pathways related to immunity and inflammation (Table 3).

### 3.2. Alterations in Gene Expression Occurring with Adipocyte Hypertrophy in Children

Obesity is not merely an accumulation of fat mass but is also associated with alterations in AT biology and function already during early childhood [3]. To address the molecular processes going on with the accumulation of AT during obesity development and progression in children in more detail, we next searched for gene expression associations with the functional AT parameters adipocyte size, leptin, adiponectin, and/or macrophage infiltration.

**Figure 2:**
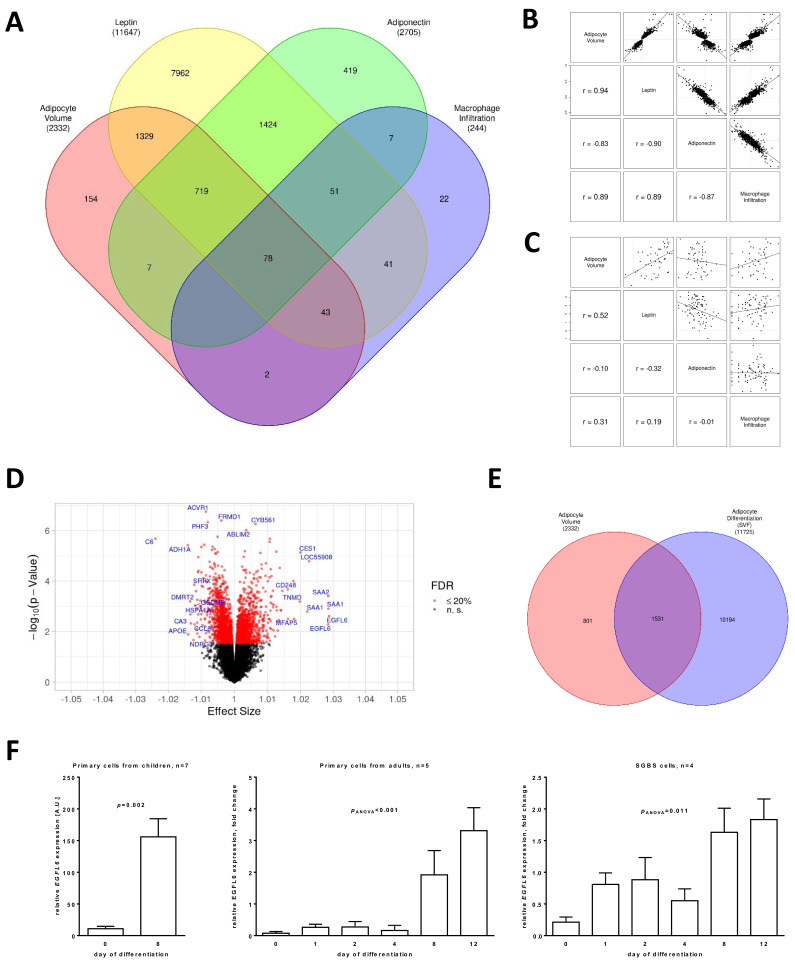
Adipose tissue dysfunction was associated with alterations in gene expression in adipocytes of children. (**A**) Venn diagram displaying the number of significant (False discovery rate FDR ≤ 20%) transcripts for gene expression analyses of adipocyte size, leptin, adiponectin, and macrophage infiltration. The numbers in brackets provide the phenotype-wise total number of significant transcripts. (**B**) Scatter plots for comparison of effect sizes between adipocyte size, leptin, adiponectin, and macrophage infiltration. Significant (FDR ≤ 20%) transcripts from gene expression analysis of adipocyte size were used as the subset for plotting and correlation to provide equal number of transcripts for all pairings. (**C**) Scatter plots for comparison of measurements between adipocyte size, leptin, adiponectin, and macrophage infiltration. (**D**) Volcano plot of the results of gene expression analysis of adipocyte size. Annotation of genes with the lowest FDR (maximum 5 genes) and strongest positive/negative effect size (maximum 10 genes each) is provided. (**E**) Venn diagram displaying the number of significant (FDR ≤ 20%) transcripts for gene expression association analyses of adipocyte size and adipocyte differentiation. The numbers in brackets provide the total number of significant transcripts for each analysis. (**F**) Bar plots (with error bars) for gene expression of *EGFL6* over time for primary cells from children (SVF) and adults as well as Simpson–Golabi–Behmel syndrome (SGBS) cells.

From the four considered phenotypes, leptin showed the highest number of significantly (FDR ≤ 0.20) associated transcripts (N = 11,647), followed by adiponectin (N = 2705), adipocyte size (N = 2332), and macrophage infiltration (N = 244, Figure 2A; Table S3). In particular, obesity-associated adipocyte hypertrophy has been closely linked to alterations in inflammatory states in AT as well as a disbalance in circulating adipokine levels, and it has been associated with an unhealthy metabolic state [4]. Interestingly, only 154 transcripts, reflecting 6.6% of all significantly regulated transcripts for adipocyte size, were associated with this phenotype but neither with leptin nor adiponectin and macrophage infiltration. Conversely, the strongest overlap of adipocyte hypertrophy-associated transcripts was with adipokine-associated transcripts with 2169 (93.0% of all transcripts significantly associated with adipocyte hypertrophy) for leptin and 804 (34.5%) for adiponectin of which 797 (34.2%) were significant for both leptin and adiponectin. In contrast, a comparably low percentage of transcripts was significantly associated with both adipocyte size and macrophage infiltration (N = 123, 5.3%). We further analysed the correlation of the effect sizes of transcripts significantly associated with each of the four parameters, adipocyte size, leptin, adiponectin, and macrophage infiltration, and observed strong positive correlations between effect sizes for adipocyte size, macrophage infiltration, and leptin, and negative correlations with adiponectin (Figure 2B). This correlation structure of effect sizes mirrors the phenotypic correlations between the parameters, though the strength of correlation was higher within gene expression than within phenotypes (Figure 2C).

Since adipocyte hypertrophy has been identified as a potential causal contributor to the development of obesity-associated metabolic diseases, we next aimed to characterise the gene expression alterations associated with adipocyte size in more detail. Of the 2332 transcripts significantly associated with adipocyte size, 1064 transcripts were upregulated, and 1268 transcripts were downregulated with increasing adipocyte hypertrophy (Figure 2D; Table S4).

To further identify genes, which might be directly involved in processes related to the regulation of adipocyte size or might act as mediators between adipocyte hypertrophy and AT dysfunction, we performed gene expression analyses of isolated SVF cells from children before and after in vitro adipocyte differentiation. To obtain a list of candidate transcripts with both association with adipocyte differentiation and adipocyte size, we combined the respective lists of significant (FDR ≤ 20%) transcripts (Figure 2E). We identified an overlap of 1531 transcripts of which 485 transcripts were regulated in the same direction and 1046 transcripts showed an inverse regulation. GO overrepresentation analyses of the top 250 significant transcripts with the strongest effect sizes revealed that these grouped mainly into pathways representing processes previously shown to be related to obesity-associated alterations in AT biology, AT function, and metabolic disease, e.g., extracellular structural organisation, angiogenesis, response to nutrient levels, response to oxygen levels, and lipid catabolic process (Table 4).

The strongest effect size from the gene expression analysis of adipocyte size was observed for EGF-like domain multiple 6 (*EGFL6*), which has been described as an extra-cellular matrix protein [20]. By measuring *EGFL6* expression in primary adipocyte progenitor cells from children and adults as well as in the adipogenesis model of SGBS cells using quantitative real-time PCR, we confirmed upregulation of *EGFL6* expression during adipocyte differentiation in vitro (Figure 2F).

### 3.3. EGFL6 Is Associated with AT Dysfunction and Metabolic Disease in Children

**Figure 3:**
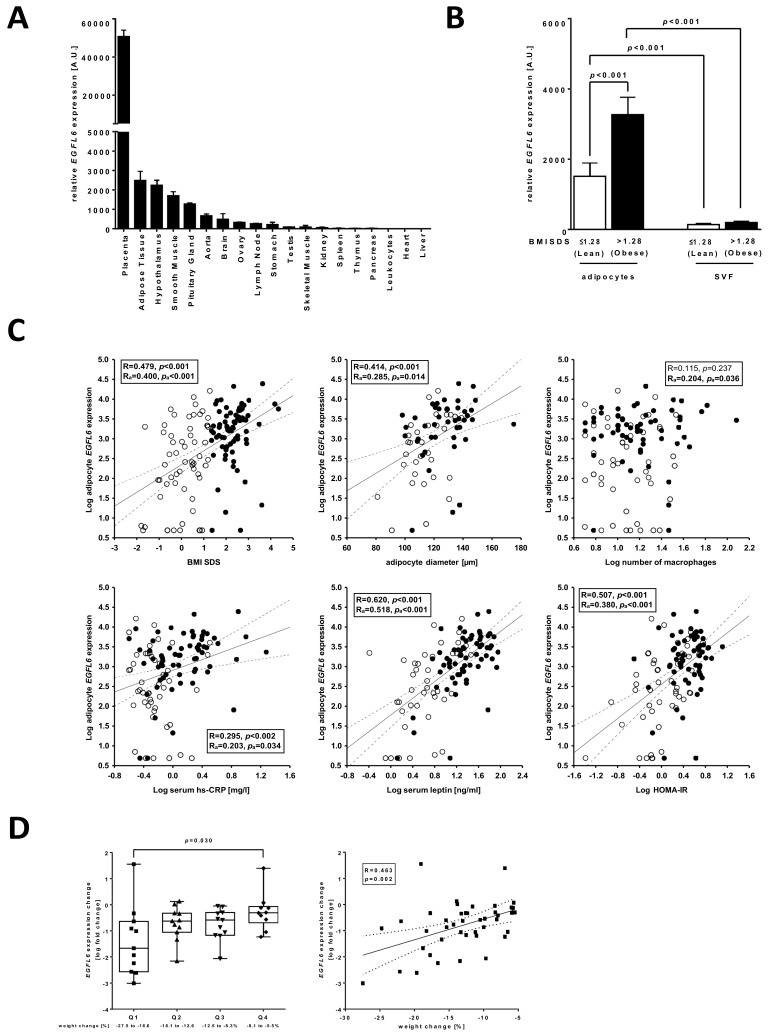
Gene expression of *EGFL6* in the adipose tissue (AT) of children and association with parameters of obesity and AT dysfunction. (**A**) *EGFL6* was quantified in a tissue panel from adults and showed the highest expression in the placenta, AT, and hypothalamus. Data are given as the mean and its standard error. (**B**) *EGFL6* gene expression was higher in isolated adipocytes compared to stromal vascular fraction (SVF) cells (bar plots with error bars, paired *t*-test, significant *p*-values are given). Both, adipocyte and SVF *EGFL6* gene expression were significantly increased in children with obesity with adipocyte expression showing the higher magnitude (2.2-fold) compared to SVF cells (1.4-fold). (**C**) Adipocyte *EGFL6* gene expression was positively associated with body mass index (standard deviation scores) (BMI SDS), adipocyte size, macrophage infiltration, high sensitive C-reactive protein (hs-CRP) and leptin serum levels, and homeostatic model assessment for insulin resistance (HOMA-IR). For analyses, children with overweight (BMI SDS > 1.28) and children with obesity (BMI SDS > 1.88) were combined in one group labelled as obese. In each scatter plot, Pearson’s correlation coefficient and the *p*-value are given two times: unadjusted (R, *p*) and adjusted for age and sex of children (R_a_, p_a_). Significant results are indicated in bold. Lean children are represented as open and children with obesity as closed circles. The solid line corresponds to the regression line while the dotted lines correspond to the respective confidence band. (**D**) The degree of weight loss in 43 adult male individuals with metabolic syndrome, who underwent 6 month long lifestyle-induced weight reduction, did not affect hs-CRP levels (N = 40), but it was significantly associated with a decrease in leptin levels (N = 24) and showed a trend towards improvement of HOMA-IR (N = 43). *EGFL6* expression in subcutaneous adipose tissue was significantly downregulated with increasing weight loss (N = 43). The box whisker plots show analyses in quartiles (Q1-Q4, different symbol for each quartile) of weight change; the whiskers indicate the minimum and maximum. Statistical analysis was performed by one-way ANOVA with Holm–Sidak’s multiple comparison test and significant *p*-values are given. In the scatter plot, the unadjusted Pearson’s correlation coefficient and *p*-value (R, *p*) are given.

In order to analyse the relevance of *EGFL6* for AT dysfunction and childhood obesity in more detail, we first measured *EGFL6* expression in different human tissues. We observed the highest expression of *EGFL6* in placenta followed by AT and hypothalamus (Figure 3A). When analysing isolated adipocytes and SVF cells derived from subcutaneous AT of children, we detected significantly increased expression of *EGFL6* in adipocytes compared to SVF cells in lean children, which was even more elevated in children with obesity (Figure 3B). In line with this, we observed significantly positive associations of adipocyte *EGFL6* expression with BMI SDS of children as well as with parameters indicative for early AT dysfunction, such as adipocyte size, macrophage infiltration into AT, serum hs-CRP and leptin levels, and metabolic disease (i.e., HOMA-IR), which was independent of the age and sex of the children (Figure 3C). However, only the associations with serum leptin levels and HOMA-IR were also independent of BMI SDS as shown by partial correlation analyses (leptin: R_adjusted_ = 0.362, p_adjusted_ < 0.001; HOMA-IR: R_adjusted_ = 0.234, p_adjusted_ = 0.016). Finally, we investigated whether the obesity-associated increase in *EGFL6* expression in AT is reversible by analysing 43 paired AT samples before and after lifestyle-induced weight loss in well-characterised male individuals. High weight loss was associated with a significant decrease in circulating leptin levels and a trend towards improvement of HOMA-IR, while hs-CRP levels were not affected by the degree of weight loss of the participants (Figure 3D). We observed a significant downregulation of *EGFL6* expression in subcutaneous AT with increasing weight loss underlining its association with obesity state (Figure 3D), while there was no significant association with hs-CRP (R = 0.085, *p* = 0.587), leptin (R = 0.326, *p* = 0.120), or HOMA-IR (R = 0.085, *p* = 0.587) change. In summary, adipocyte *EGFL6* expression was related to obesity, AT dysfunction, and early signs of metabolic disease in children and obesity-related alterations in *EGFL6* expression can be reversed by weight loss.

## 4. Discussion

Here, we investigated the association of genome-wide gene expression profiles of AT samples and in vitro differentiation experiments with parameters of the early development of adipocyte hypertrophy and AT dysfunction in children.

Obesity manifests early in life and childhood obesity is a strong risk factor for the development of obesity-associated cardiovascular and metabolic diseases and premature death in adulthood [9,21]. Hence, it is of particular interest to understand the molecular processes associated with early AT expansion during childhood obesity.

Thus far, there are only a few studies on genome-wide expression profiles in AT samples of children, and none of them addressed the association of gene regulation with adipocyte hypertrophy and AT dysfunction. Tam et al. compared transcriptomic profiles of visceral and subcutaneous AT samples in children and showed that subcutaneous AT had an overrepresentation of transcripts related to cell growth and development, while visceral AT showed an expression profile related to immune and inflammatory responses, which they hypothesised to contribute to increased metabolic and cardiovascular risk in later life [22]. This assumption was further underlined by results from Aguilera et al., showing that genes associated with lipid metabolism, oxidative stress, extracellular matrix (ECM) organisation, and inflammation are upregulated in visceral AT of children with obesity compared to normal weight children [23]. Analyses of genome-wide expression data of subcutaneous AT samples from eight lean children and seven children with overweight and obesity in a more recent study showed that differentially expressed genes were enriched in the GO terms related to extracellular space and immune and inflammatory response [24]. Similar to these previous studies, we detected an overrepresentation of metabolic pathways and pathways related to ECM organisation and inflammation regulated with increasing BMI SDS in subcutaneous AT samples of children. Interestingly, when we analysed adipocytes and SVF cells separately, there were clear differences between the two AT fractions with adipocytes showing a regulation of pathways related to metabolism and ECM organisation and SVF cells showing a rather specific regulation of inflammatory pathways, indicating the presence of immune cells. In this context, we and others have previously shown that childhood obesity is already associated with an infiltration of macrophages and a formation of crown-like structures in AT [3,25].

Furthermore, based on protein–protein interaction network analyses of differentially expressed genes in the AT of children with normal weight or overweight and obesity, Li et al. suggested matrix metalloproteinase 9 (*MMP9*) and acetyl-CoA carboxylase β (*ACACB*) as marker genes for childhood obesity [24]. In line with this, we identified *MMP9* as significantly downregulated and *ACACB* as significantly upregulated in AT, isolated adipocytes, and SVF cells with increasing BMI SDS of children (Table S2). However, neither *MMP9* nor *ACACB* were regulated with adipocyte hypertrophy (Table S3), indicating that the expression levels of these genes are associated with different parameters of AT biology such as an inflammatory state.

We identified *EGFL6* as the most strongly regulated gene with the increasing size of adipocytes in children, indicative for being involved in processes related to adipocyte hypertrophy and AT dysfunction. EGFL6 belongs to the EGF repeat superfamily of proteins, which are secreted proteins and involved in a wide range of biological functions including, proliferation, differentiation, migration, adhesion, and apoptosis [26,27]. Based on its protein structure, EGFL6 has been proposed to be an extra-cellular matrix protein with a predicted function in cell adhesion [20]. *EGFL6* is highly conserved in vertebrates and has been described to be expressed in a variety of fetuses and adults [28,29,30]. Similar to other members of the EGF repeat superfamily, EGFL6 has been shown to induce angiogenesis in different biological contexts. For example, during bone development and remodelling, EGFL6 acts in a paracrine manner, being secreted from osteoblastic-like cells and acting on endothelial cells triggering cell proliferation, migration, and capillary tube formation. Furthermore, *EGFL6* has been shown to be expressed in some cases of hyperplasia, such as ovarian cancer and meningioma [31,32], and it is particularly highly expressed in tumour-associated endothelial cells compared to other endothelial cells and is involved in tumour angiogenesis [33].

Apart from this, EGFL6 has been identified as an AT-secreted protein in adult humans, where it has been implicated in the regulation of adipose progenitor cell proliferation and obesity-related AT expansion, while a link to obesity-related adipocyte hypertrophy, AT dysfunction, and metabolic impairment was proposed but not analysed in this study [20].

By analysing a tissue panel obtained from adult humans, we detected the strongest expression of *EGFL6* in the placenta, which was 25-fold higher compared to AT, which showed the second strongest expression of all tissues analysed. Interestingly, during pregnancy, the placenta undergoes extensive tissue remodelling in terms of the reconstruction of the ECM and angiogenesis [34]. Similar processes occur in AT with increasing adipocyte hypertrophy during development and progression of obesity [30] and, in fact, AT has been described as one of the tissues in the human body with the highest angiogenic capacities [35], hence, indicating a role for *EGFL6* in the regulation of processes related to angiogenesis.

One strength of our study is that compared to previous studies, we analysed genome-wide expression profiles in a large cohort of children, while most previous studies assessing molecular processes related to AT expansion and AT (dys)function were performed in adults who present at later stages of the disease and often already suffer from obesity-associated comorbidities. Furthermore, the exposure time to drug treatments, environmental compounds, or diets that might affect AT biology and function was smaller in children compared to adults. A second strength was that we not only analysed whole AT samples but also isolated adipocytes and SVF cells, which might provide more detailed insight into the regulation of obesity-related processes specific to these subfractions of AT. However, we are aware that SVF cells still represent a mixture of different cell types, including endothelial and adipose progenitor cells, fibroblasts, and immune cells [36], and that regulated pathways might reflect differences in cellular composition.

In conclusion, we provide evidence for early obesity-associated alterations in the expression of genes involved in metabolic pathways and pathways related to ECM organisation and inflammation in AT as well as isolated adipocytes and SVF cells of children. Moreover, we identify *EGFL6* as the gene most strongly positively associated with adipocyte hypertrophy and showed that its expression is related to early signs of AT dysfunction and metabolic disease, indicating a potential role in the pathogenesis of these processes.

## Figures and Tables

**Table 1:** Cohort characteristics of probands and samples included in transcriptome analyses.

Trait	Transcriptome Subset Analysed	Sample Size, N	Mean (SD)	Range
**Whole cohort available for transcriptome analysis**				
Sex (male/female)		317 (191/126)	-	-
Age (years)		317	9.6 (5.5)	0.1–20.7
**Transcriptome analysis related to BMI SDS of children**				
BMI SDS	AT	306	0.7 (1.5)	−3.2–4.3
	Adipocytes	117	1.3 (1.4)	−2.5–4.3
	SVF	113	1.3 (1.4)	−1.8–4.3
**Transcriptome analysis related to parameters of AT dysfunction**				
Adipocyte size (µm)	Adipocytes	62	119.1 (14.0)	90.9–146.2
Leptin (ng/mL)	Adipocytes	100	21.7 (21.0)	0.4–99.0
Adiponectin (mg/L)	Adipocytes	98	7.8 (5.9)	1.7–43.8
Macrophage infiltration (number/100 adipocytes)	Adipocytes	94	13.3 (16.9)	0–115

For BMI SDS analyses, all three tissues (i.e., AT, adipocytes, and SVF) were considered, whereas for adipocyte size, leptin, adiponectin, and macrophage infiltration, we only considered gene expression measurements in adipocytes. BMI SDS, body mass index standard deviation score; AT, adipose tissue; SVF, stromal vascular fraction.

**Table 2:** Cohort characteristics of probands and samples included in the qRT-PCR analysis.

		Lean			Obese		
	N	Mean ± SEM	Range	N	Mean ± SEM	Range	*p*
Male/Female	59	27/32 (45.8)	-	73	34/39 (46.6)	-	0.926 ^a^
(% male)							
Age (years)	59	10.4 ± 0.6	1.1–18.3	73	13.3 ± 0.3	4.8–18.4	<0.001
PH	50	2.4 ± 0.2	1–6	63	3.4 ± 0.2	1–6	0.002
BMI SDS	59	0.1 ± 0.1	−1.8–1.2	73	2.3 ± 0.1	1.3–4.3	<0.001
Adipocyte size (µm)	32	114.3 ± 2.3	80.9-138.8	43	127.2 ± 2.4	98.0–174.8	<0.001
Macrophages per 100 adipocytes	49	8.8 ± 1.1	0-29	59	15.8 ± 2.4	0–115	0.013 ^b^
Number of children with CLS (%)	49	7 (14.3%)		59	31 (52.5%)		<0.001 ^a^
Adiponectin (mg/L)	45	9.0 ± 1.0	1.6–43.8	63	5.6 ± 0.3	1.7–15.1	<0.001 ^b^
Leptin (ng/mL)	42	7.5 ± 1.2	0.4–28.2	64	29.4 ± 2.7	1.3–89.1	<0.001 ^b^
HOMA-IR	48	1.5 ± 0.2	0.04–5.6	62	3.6 ± 0.3	0.3–12.7	<0.001 ^b^

PH, pubertal stage according to pubic hair; BMI SDS, body mass index standard deviation score; CLS, crown-like structures; HOMA-IR, homeostasis model assessment of insulin resistance. ^a^ For sex and occurrence of CLS, statistical significance was determined by the chi-squared test; ^b^ statistical analyses were performed for log-transformed parameters.

**Table 3:** Results of GO overrepresentation analyses for transcripts related to BMI SDS.

Gene Set	Description	Size	Enrichment Ratio	FDR
**Adipose tissue**				
GO:0050900	leukocyte migration	413	3.9	8.2 × 10^−5^
GO:1901615	organic hydroxy compound metabolic process	494	3.6	8.2 × 10^−5^
GO:0008202	steroid metabolic process	302	4.3	3.0 × 10^−4^
GO:0006898	receptor-mediated endocytosis	279	4.1	2.0 × 10^−3^
GO:0044282	small molecule catabolic process	419	3.3	3.0 × 10^−3^
GO:0043062	extracellular structural organisation	392	3.3	3.7 × 10^−3^
GO:0002446	neutrophil-mediated immunity	484	3.0	3.7 × 10^−3^
GO:0036230	granulocyte activation	488	3.0	3.7 × 10^−3^
GO:0060326	cell chemotaxis	285	3.7	5.1 × 10^−3^
GO:0062012	regulation of small molecule metabolic process	338	3.4	6.7 × 10^−3^
**Adipocytes**				
GO:0043062	extracellular structural organisation	392	6.2	7.6 × 10^−13^
GO:0006898	receptor-mediated endocytosis	279	4.1	4.1 × 10^−3^
GO:0050900	leukocyte migration	413	3.3	4.1 × 10^−3^
GO:0007492	endoderm development	72	7.9	5.8 × 10^−3^
GO:0071559	response to transforming growth factor beta	234	4.2	5.8 × 10^−3^
GO:0006638	neutral lipid metabolic process	118	5.5	1.2 × 10^−2^
GO:0048771	tissue remodelling	151	4.8	1.2 × 10^−2^
GO:0002526	acute inflammatory response	153	4.8	1.2 × 10^−2^
GO:0016042	lipid catabolic process	310	3.4	1.2 × 10^−2^
GO:0050673	epithelial cell proliferation	359	3.2	1.2 × 10^−2^
**Stromal vascular fraction cells**				
GO:0050900	leukocyte migration	413	6.2	1.9 × 10^−13^
GO:0036230	granulocyte activation	488	5.6	1.9 × 10^−13^
GO:0002446	neutrophil-mediated immunity	484	5.1	3.6 × 10^−11^
GO:0002237	response to molecule of bacterial origin	326	5.6	1.3 × 10^−8^
GO:0002694	regulation of leukocyte activation	473	4.6	1.6 × 10^−8^
GO:0071216	cellular response to biotic stimulus	221	6.7	2.3 × 10^−8^
GO:0002521	leukocyte differentiation	489	4.4	2.3 × 10^−8^
GO:0060326	cell chemotaxis	285	5.8	2.6 × 10^−8^
GO:0050727	regulation of inflammatory response	358	5.1	3.7 × 10^−8^
GO:0006909	phagocytosis	234	6.4	3.9 × 10^−8^

From all significantly (False discovery rate FDR ≤ 5%) associated transcripts, the 250 transcripts with the highest absolute effect sizes were considered. BMI SDS, body mass index standard deviation score; GO, gene ontology.

**Table 4:** Results of GO overrepresentation analyses for transcripts related to adipocyte size and adipocyte differentiation.

Gene Set	Description	Size	Enrichment Ratio	FDR
**Adipocytes**				
GO:0043062	extracellular structural organisation	392	4.2	5.3 × 10^−5^
GO:0001525	angiogenesis	476	3.5	5.8 × 10^−4^
GO:0090287	regulation of cellular response to growth factor stimulus	252	4.6	6.9 × 10^−4^
GO:0042326	negative regulation of phosphorylation	417	3.4	2.8 × 10^−3^
GO:0031667	response to nutrient levels	474	3.1	3.2 × 10^−3^
GO:0050673	epithelial cell proliferation	359	3.4	4.6 × 10^−3^
GO:0070482	response to oxygen levels	326	3.5	5.4 × 10^−3^
GO:0033002	muscle cell proliferation	180	4.6	7.4 × 10^−3^
GO:1902532	negative regulation of intracellular signal transduction	486	2.9	8.5 × 10^−3^
GO:0016042	lipid catabolic process	310	3.5	9.1 × 10^−3^

From all transcripts significantly associated with both adipocyte size and adipocyte differentiation (False discovery rate FDR ≤ 20%), the 250 transcripts with the highest absolute effect sizes were considered. GO, gene ontology.

## Data Availability

The transcriptomics data analysed in this study are available upon request from the corresponding author. The data are not publicly available due to the fact of privacy reasons.

## Supplementary Materials

The following supporting information can be downloaded at: https://www.mdpi.com/article/10.3390/ijms23084349/s1.
