# Supplementary material for: Transcriptome Analyses of Adipose Tissue Samples Identify EGFL6 as a Candidate Gene Involved in Obesity-Related Adipose Tissue Dysfunction in Children
Source: Int J Mol Sci. 2022 Apr 14;23(8):4349. doi: 10.3390/ijms23084349 (PMC9033114; doi:10.3390/ijms23084349)
Supplement: Supplementary file 1 [file ijms-23-04349-s001.zip › Table S1 - Primer and probe sequences for qPCR.pdf]

Table S1. Primer and probe sequences for quantitative *real-time* PCR.

Symbol	Gene name	Forward Primer	Reverse Primer	Probe
EGFL6	Epidermal growth factor-like domain multiple 6	AAAGACATTGGCCGA TTGAAAC	CAGCCGGTAATCAAA GAGCAA	CTACCTGACCTGCAACCCC AAAGCAACTT
ACTB	β-actin	TGAGCGCGGCTACAG CTT	CCTTAATGTCACGCAC GATTT	ACCACCACGGCCGAGCGG
TBP	TATA-box-binding protein	TTGTAAACTTGACCTA AGACCATTGC	TTCGTGGCTCTCTTAT CCTCATG	AACGCCGAATATAATCCC AAGCGGTTTG
HPRT	hypoxanthine-guanine phosphoribosyl-transferase	GGCAGTATAATCCAA AGATGGTCAA	GTCTGGCTTATATCCA ACACTTCGT	CAAGCTTGCTGGTGAAAA GGACCCC

Forward and reverse primers are given in 5´-3´direction. Probes were labelled with the reporter 5’-FAM or 5’-HEX for *TBP* and the quencher 3’-TAMRA.
